# Cultivated and wild *Pleurotus ferulae* ethanol extracts inhibit hepatocellular carcinoma cell growth via inducing endoplasmic reticulum stress- and mitochondria-dependent apoptosis

**DOI:** 10.1038/s41598-018-32225-4

**Published:** 2018-09-18

**Authors:** Yi Yang, Pengfei Yuan, Xianxian Wei, Changshuang Fu, Jinyu Li, Weilan Wang, Xinhui Wang, Yijie Li, Jinyao Li

**Affiliations:** 1 Xinjiang Key Laboratory of Biological Resources and Genetic Engineering, College of Life Science and Technology, Xinjiang University, Urumqi, 830046 China; 2 College of Life Science, Xinjiang Normal University, Urumqi, 830054 China; 3 Affliated Tumor Hospital of Xinjiang Medical University, Urumqi, 830011 China

## Abstract

*Pleurotus ferulae* is a kind of editable mushroom and has various biological functions such as antitumor, antioxidation and immunoregulation. Wild *P. ferulae* was successfully domesticated but the antitumor function and mechanisms of cultivated and wild *P. ferulae* need to be compared and explored. Here, we prepared cultivated and wild *P. ferulae* ethanol extracts (PFEE-C and PFEE-W) and compared their antitumor effect on hepatocellular carcinoma. Our data showed that PFEE-C and PFEE-W significantly inhibited the growth of H22 and HepG2 cells through induction of apoptosis. PFEE-W exhibited higher antitumor activity than PFEE-C. Both PFEE-C and PFEE-W induced endoplasmic reticulum (ER) stress characterized by the up-regulated levels of phosphorylated JNK, cleaved caspase-12 and HSP70, and mitochondrial dysfunction characterized by the reduction of mitochondrial membrane potential and the release of cytochrome c, which promoted the cleavage of caspase-3, -7, -9 and PARP. Moreover, PFEE-C and PFEE-W significantly increased ROS generation in H22 cells and suppressed H22 cell migration through reducing the levels of matrix metalloproteinase -2 and -9. Further, PFEE-C inhibited H22 tumor growth in mouse model and improved the survival of tumor mice. These results indicated that PFEE-C and PFEE-W could inhibit hepatocellular carcinoma cell growth through ER stress- and mitochondria-dependent apoptotic pathways.

## Introduction

Liver cancer, which consists predominantly of hepatocellular carcinoma (HCC), ranks the sixth for cancer incidence and the fourth for cancer mortality worldwide^1^. The highest incidence and mortality rates of HCC were found in East Asia and central sub-Saharan Africa^1^, which resulted from chronic infection with hepatitis virus and other factors including food- and water-borne carcinogens^2^. In 2015, the estimated new liver cancer cases are 466,100 and the estimated deaths are 422,100 in China^3^. Currently, the treatments of liver cancer include surgery, targeted therapy, radiotherapy, chemotherapy, or their combinations^4–8^. However, the clinical efficacy is still unsatisfied. It definitively needs to develop safe and effective drugs for treating liver cancer.

Accumulating evidence has shown that edible mushrooms have many biological activities and have been used as a source of natural medicine and functional food^9,10^. *Pleurotus ferulae* is an edible mushroom and grows on the living rhizome trunks of *Ferula asafoetida* in the Gobi desert, which is mainly distributed in Xinjiang, China^11^. Several studies including ours have reported that *P. ferulae* has anti-tumor, anti-microbial, anti-oxidant and immunomodulatory functions^11–16^. It has been shown that the cytotoxicity of *P*. *ferulae* ethanol extract is higher than that of hot water extract on several human cancer cell lines and can induce the synergistic effects on the TRAIL-induced apoptosis in A549 cells^17^. Our previous study also showed that *P. ferulae* ethanol extract (PFEE) inhibited the growth of melanoma cell line B16F10 *in vitro* and *in vivo* through induction of cell cycle arrest and mitochondria-mediated apoptosis^11^. Due to the limit resource and important values in nutrition and pharmacology, wild *P. ferulae* was successfully domesticated by Xinjiang Institute of soil biological desert in 1990. Whether wild and/or cultivated *P. ferulae* have antitumor effect on HCC and the difference of their antitumor effect on HCC are still elusive.

In this study, we prepared ethanol extracts of cultivated and wild *P. ferulae* and named as PFEE-C and PFEE-W, respectively. The antitumor effects of PFEE-C and PFEE-W on HCC were detected and compared in H22 and HepG2 cells. We found that both PFEE-C and PFEE-W could inhibit the growth of H22 and HepG2 cells through induction of apoptosis, which was mediated by mitochondria-dependent and endoplasmic reticulum (ER) stress-dependent pathways in H22 cells. The results indicated that PFEE might be used to develop antitumor drugs against HCC.

## Results

### PFEE-C and PFEE-W inhibit the growth of H22 and HepG2 cells *in vitro*

**Figure 1:**
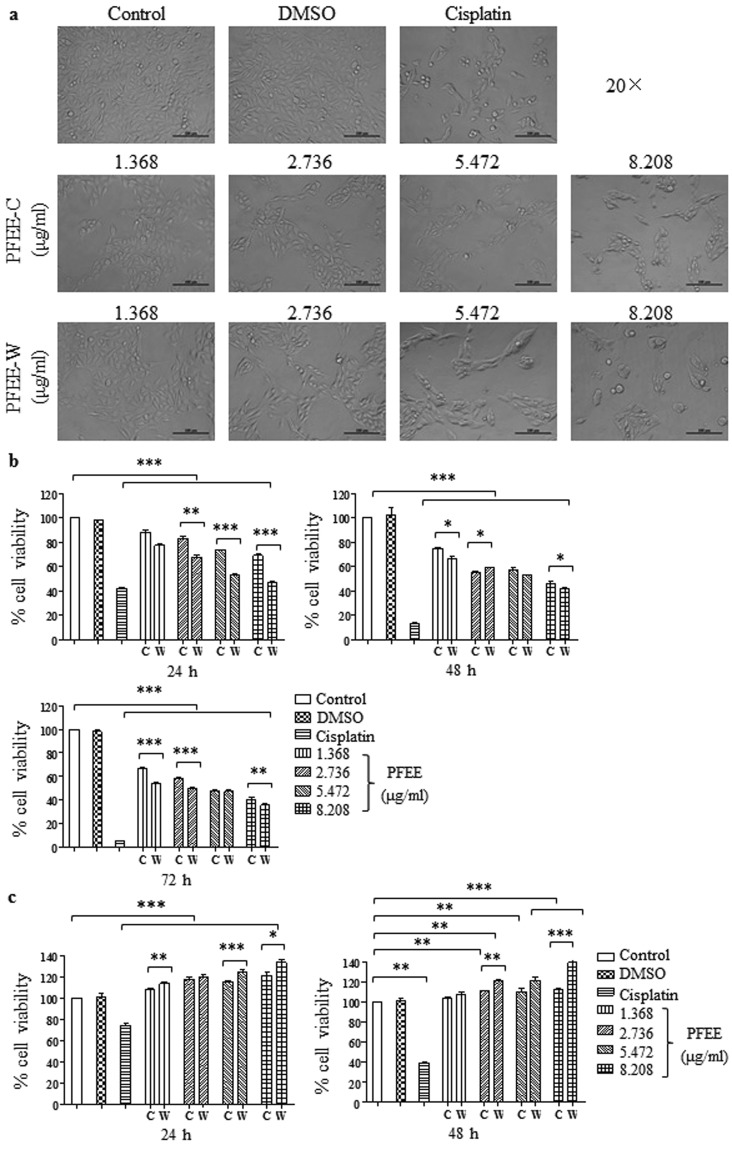
Effects of PFEE-C and PFEE-W on the proliferation of H22 cells and splenocytes *in vitro*. (**a**) The morphological changes of H22 cells after PFEE-C and PFEE-W treatment for 24 h. (**b**) The viability of H22 cells after PFEE-C and PFEE-W treatment for 24, 48 and 72 h, respectively. (**c**) The proliferation of splenocytes after PFEE-C and PFEE-W treatment for 24, 48 and 72 h, respectively. Data are from 3 independent experiments and analyzed by ANOVA. **p < 0.01; ***p < 0.001 compared to untreated group.

The ethanol extractions of cultivated and wild *P. ferulae* were prepared and named as PFEE-C and PFEE-W. Their flavonoid contents are 1.37% and 1.5%, respectively. To investigate the antitumor effect of PFEE, H22 and HepG2 cells were treated with different concentrations of PFEE-C and PFEE-W according to their flavonoid contents. After 24 h, the morphology of H22 cells was observed by microscope and it was significantly changed by PFEE-C and PFEE-W treatment in a dose-dependent manner (Fig. 1a). The similar changes of cell morphology were observed in HepG2 cells (Supplemental Fig. 1a). The viability of H22 and HepG2 cells was measured by MTT assay at the indicated time points. As shown in Fig. 1b, both PFEE-C and PFEE-W were significantly reduced the viability of H22 cells in a dose- and time-dependent manner compared to control (p < 0.001). Moreover, the inhibitory activity of PFEE-W was significantly higher than that of PFEE-C on H22 cells after 24 h and 72 h (Fig. 1b). Similar cytotoxicity of PFEE-C and PFEE-W were observed in HepG2 cells (Supplemental Fig. 1b). The inhibition rates of 5.472 and 8.208 μg/ml flavonoids in PFEE-C and PFEE-W on H22 cells were higher than 50% and 60%, respectively, after 72 h treatment. We also detected the effect of PFEE on the proliferation of murine splenocytes. The results showed that both PFEE-C and PFEE-W significantly increased the proliferation of splenocytes (p < 0.01). PFEE-W showed stronger activity on splenocyte proliferation than PFEE-C at 8.208 μg/ml flavonoids (Fig. 1c). These results suggested that PFEE-C and PFEE-W inhibited H22 and HepG2 cell growth in a dose-dependent and time-dependent manner, but they had no cytotoxic effect on splenocytes.

### PFEE-C and PFEE-W induce apoptosis of H22 and HepG2 cells

**Figure 2:**
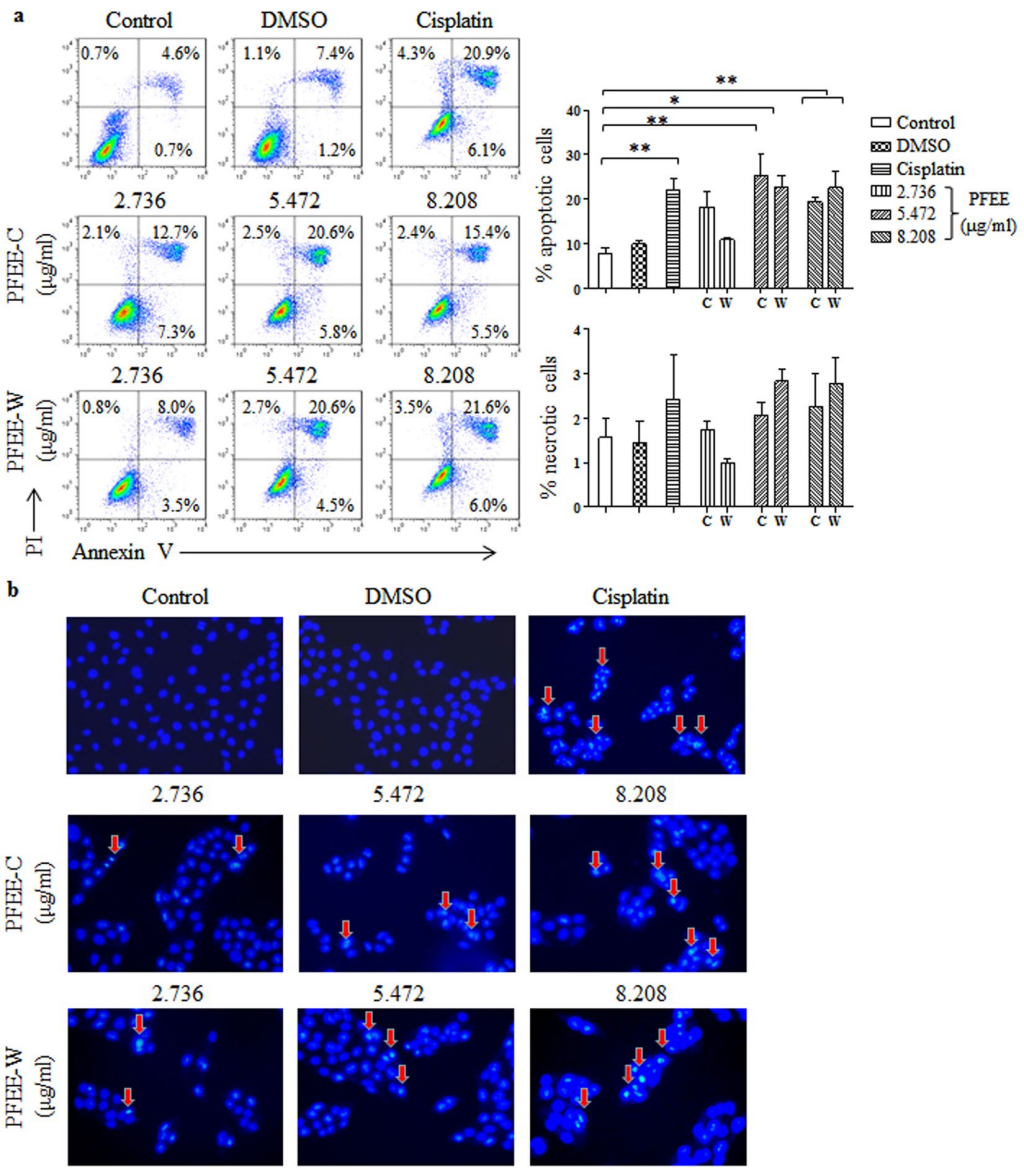
The apoptosis of H22 cells induced by PFEE-C and PFEE-W treatment. Different concentrations of PFEE-C and PFEE-W were used to treat H22 cells for 24 h. (**a**) The apoptosis and necrosis of H22 cells were analyzed by flow cytometry. The individual dot plots were shown in left panels and the summary data were shown in right panels. (**b**) The nuclear morphology of H22 cells. The above H22 cells were stained with Hoechst 33258 and observed by inverted fluorescence microscopy. The arrows indicated the chromosomal condensation. Data are from 3 independent experiments and analyzed by ANOVA. *p < 0.05; **p < 0.01 compared to untreated group.

Next, we detected whether PFEE inhibited the growth of H22 and HepG2 cells by induction of apoptosis. H22 and HepG2 cells were treated with different concentrations of PFEE-C and PFEE-W for 24 h and stained with Annexin V-FITC and PI. Samples were analyzed by flow cytometry. Compared with the untreated group, the frequencies of apoptotic H22 cells were significantly increased (p < 0.05) but the frequencies of necrotic H22 cells were not significantly changed upon PFEE-C and PFEE-W treatment (Fig. 2a). For HepG2 cells, PFEE-C and PFEE-W significantly induced necrosis but mainly induced apoptosis (Supplemental Fig. 2a).

The apoptosis of H22 and HepG2 cells was further measured by hoechst 33258 staining after PFEE-C and PFEE-W treatment for 24 h. The nuclear morphology was observed by inverted fluorescence microscope. As shown in Fig. 2b, the untreated and DMSO treated H22 cells were homogeneously stained, however, PFEE-C and PFEE-W treated H22 cells showed chromatin condensation and fragmentation in a dose-dependent manner, which was similar with cisplatin treated H22 cells. Similar changes of nuclei were observed in HepG2 cells (Supplemental Fig. 2b). These results indicated that PFEE-C and PFEE-W induced apoptosis of H22 and HepG2 cells.

### PFEE-C and PFEE-W induce cell cycle arrest in H22 cells

**Figure 3:**
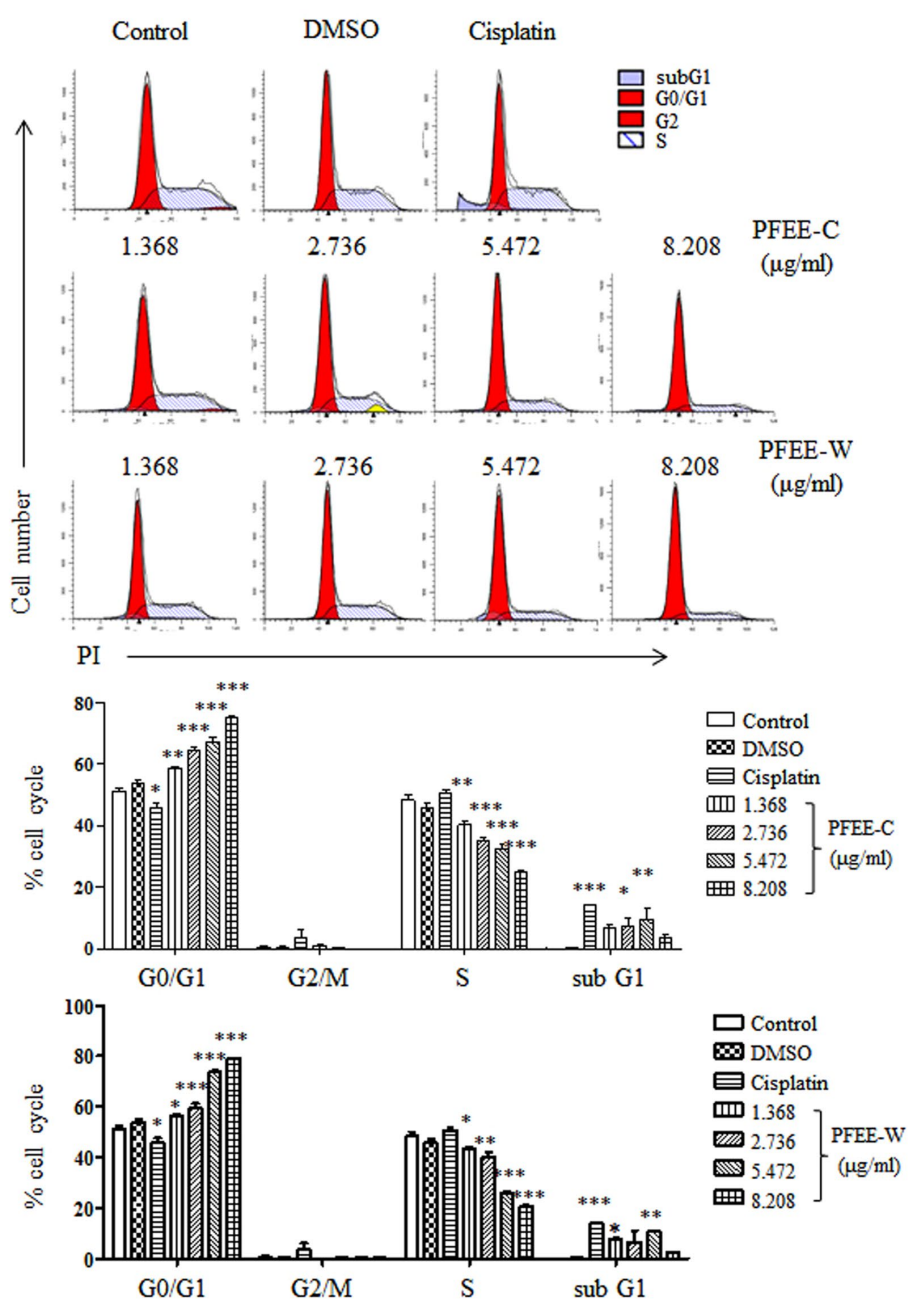
Cell cycle distribution in H22 cells upon PFEE-C and PFEE-W treatment. H22 cells were treated with different concentrations of PFEE-C and PFEE-W for 24 h. After PI staining, cell cycle distribution was analyzed by flow cytometry. Data are from 3 independent experiments and analyzed by ANOVA. *p < 0.05; **p < 0.01; ***p < 0.001 compared to untreated group.

Due to PFEE-C and PFEE-W induced chromatin condensation and fragmentation in H22 cells, we detected whether PFEE-C and PFEE-W affected cell cycle in H22 cells. H22 cells were treated with different concentrations of PFEE for 24 h and stained with PI. Cell cycle distribution in H22 cells was analyzed by flow cytometry. After PFEE-C and PFEE-W treatment, an accumulation of H22 cells at G0/G1-phase was observed in a dose-dependent manner and its frequencies increased from 51.1% in untreated group to 75% in PFEE-C and 79.1% in PFEE-W at 8.208 μg/ml flavonoids, respectively (Fig. 3). These results indicated that PFEE-C and PFEE-W induced G0/G1-phase arrest in H22 cells.

### PFEE-C and PFEE-W decrease mitochondrial membrane potential (Δψ_m_) in H22 cells

**Figure 4:**
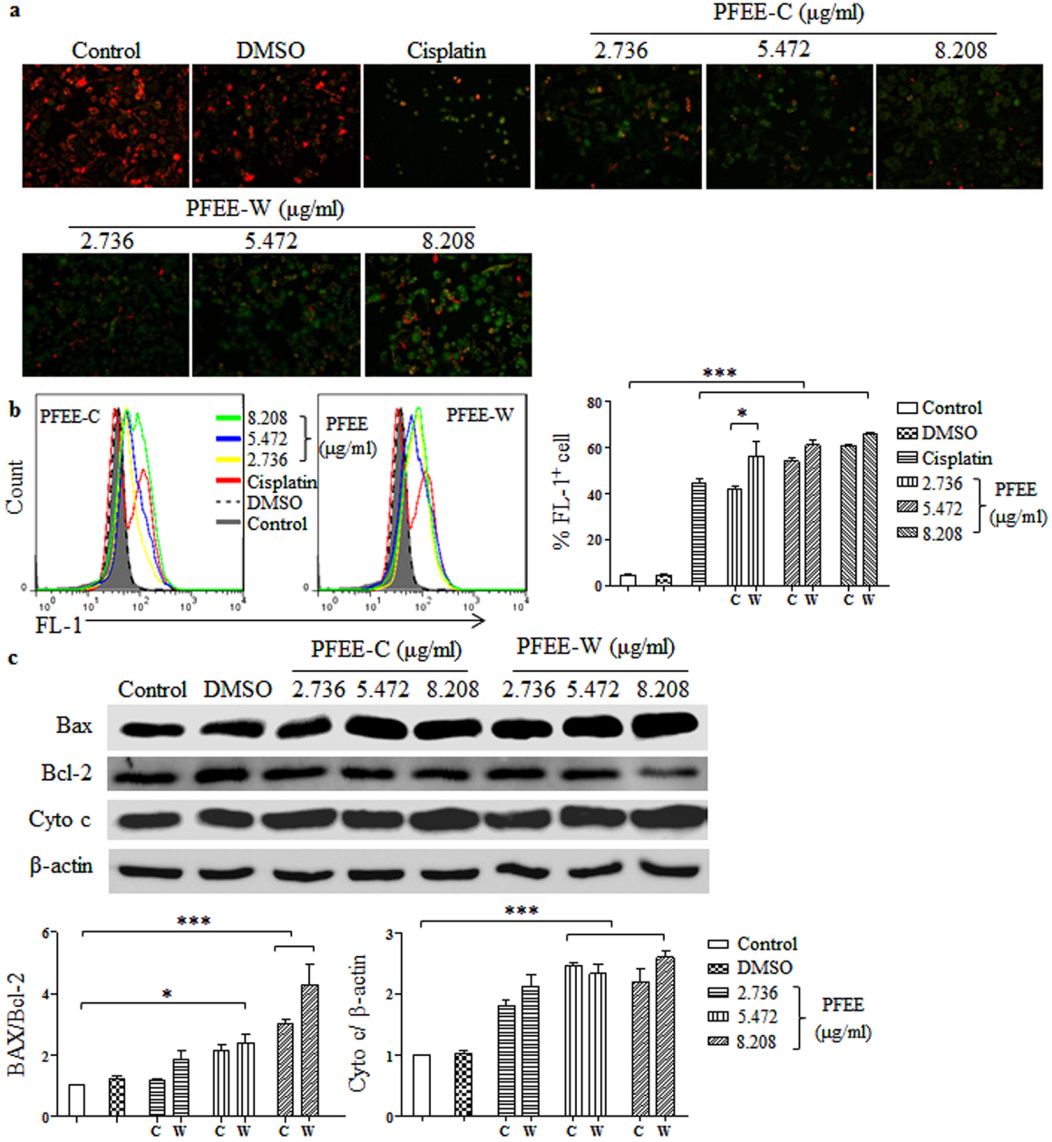
The effects of PFEE-C and PFEE-W on Δψ_m_ and intracellular ROS generation in H22 cells. H22 cells were treated with different concentrations of PFEE-C and PFEE-W. After 48 h, cells were stained with JC-1 and the fluorescence changes were observed using inverted fluorescence microscopy (**a**) and analyzed by flow cytometry (**b**). (**c**) After 24 h, proteins were isolated and the levels of Bax, Bcl-2 and cytochrome (cyto) c were detected by Western blot. Cropped blots are shown and full-length blots are included in the Supplementary Information. Grayscale scanning data were obtained by Image J. The ratios of Bax/Bcl-2 and cyto c/β-actin were shown in lower panels.

To investigate whether the apoptosis of H22 cells induced by PFEE-C and PFEE-W was mediated by the mitochondria-dependent pathway, cells were treated with PFEE-C and PFEE-W for 48 h. After JC-1 staining, samples were observed by inverted fluorescence microscopy and analyzed by flow cytometry. We observed that the red fluorescence was changed to green fluorescence upon PFEE-C and PFEE-W treatment (Fig. 4a). Consistently, FL-1^+^ cells were significantly increased (p < 0.001, Fig. 4b), suggesting that Δψ_m_ in H22 cells was reduced by PFEE-C and PFEE-W treatment. In addition, PFEE-W showed higher activity than PFEE-C in the reduction of Δψ_m_.

The B cell lymphoma 2 (BCL-2) protein family including Bax and Bcl-2 was involved in the regulation of mitochondrial membrane integrity^18,19^. After PFEE-C and PFEE-W treatment for 24 h, total proteins of H22 cells were isolated to detect the levels of Bax and Bcl-2 by Western blot. As shown in Fig. 4c, the levels of Bax and Bcl-2 were up-regulated and down-regulated, respectively, by PFEE-C and PFEE-W treatment. The ratio of Bax/Bcl-2 was significantly increased in PFEE-C and PFEE-W treated cells compared with untreated cells, which was consistent with the reduction of Δψ_m_. Consequentially, the release of cytochrome c was significantly increased upon PFEE-C and PFEE-W treatment (p < 0.001, Fig. 4c). These results suggested that PFEE-W and PFEE-C induced Δψ_m_ reduction.

### PFEE-C and PFEE-W promote caspase and poly ADP-ribose polymerase (PARP) processing in H22 cells

**Figure 5:**
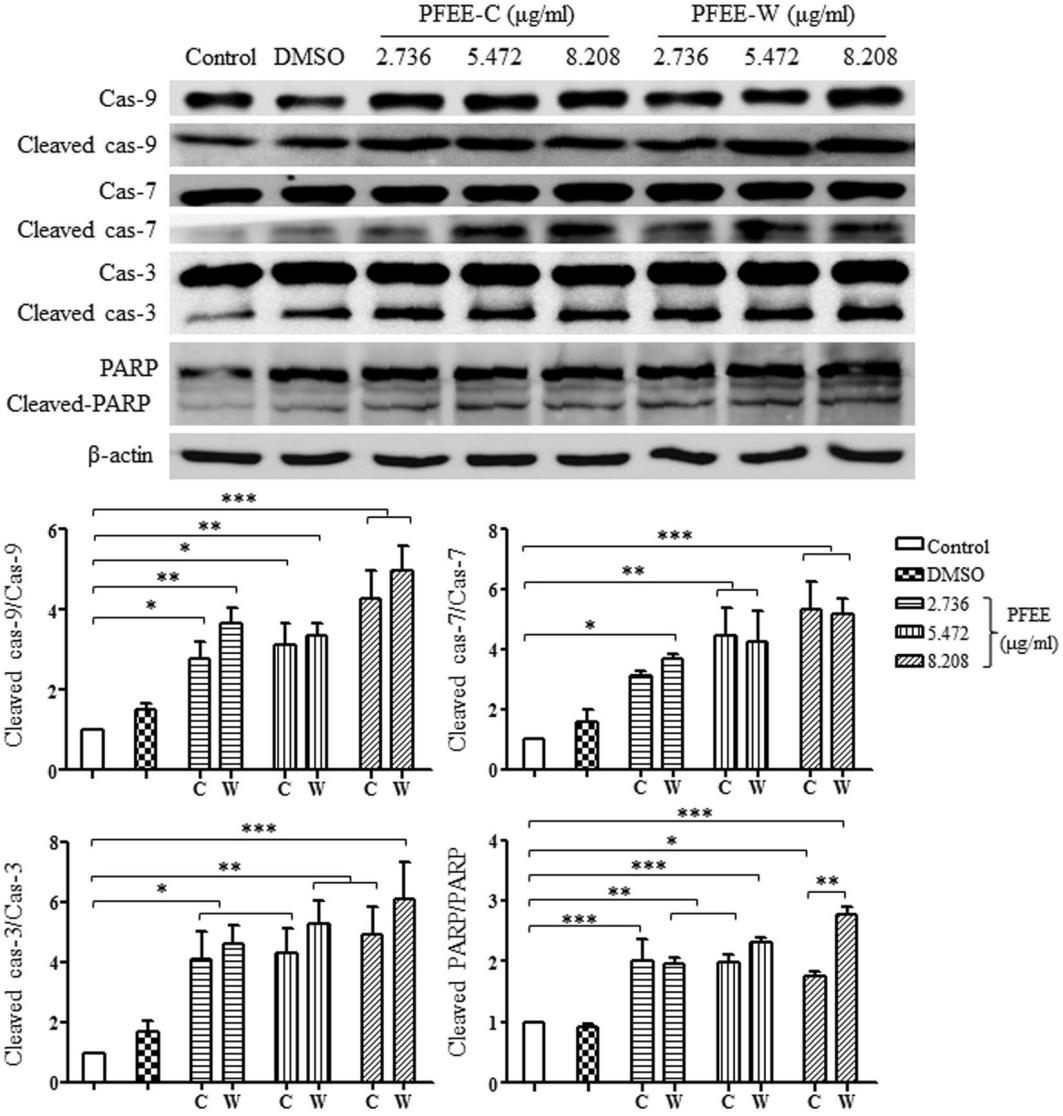
PFEE-C and PFEE-W activated caspases in H22 cells. H22 cells were treated with different concentrations of PFEE-C and PFEE-W. After 24 h, proteins were isolated and the levels of cleaved-caspases and -PARP were detected by Western blot. Cropped blots are shown and full-length blots are included in the Supplementary Information. Grayscale scanning data were obtained by Image J. The ratios of cleaved-caspases/caspases and cleaved-PARP/PARP were shown in lower panels. Data are from 3 independent experiments and analyzed by ANOVA. *p < 0.05; **p < 0.01; ***p < 0.001 compared to untreated group.

The release of cytochrome c can activate the initiator caspase-9. Therefore, the levels of initiator and effector caspases were detected by Western blot. After treatment with PFEE-C and PFEE-W for 24 h, proteins of H22 cells were prepared for analyzing the levels of caspase (cas)-3, cleaved cas-3, cas-7, cleaved cas-7, cas-9 and cleaved cas-9 (Fig. 5). We found that PFEE-C and PFEE-W significantly increased the ratios of cleaved cap-3/cas-3, cleaved cap-7/cas-7 and cleaved cap-9/cas-9 (p < 0.05). We also observed that the ratio of cleaved PARP/PARP was significantly increased in both PFEE-C and PFEE-W treated groups compared to untreated group (p < 0.05), suggesting that DNA damage induced by PFEE-C and PFEE-W cannot be effectively repaired. At 8.208 μg/ml flavonoids, PFEE-W showed higher ratio of cleaved PARP/PARP than that of PFEE-C. These data suggest that PFEE-C and PFEE-W induced the apoptosis in H22 cells through mitochondria-dependent pathway.

### PFEE-C and PFEE-W induce endoplasmic reticulum (ER) stress in H22 cells

**Figure 6:**
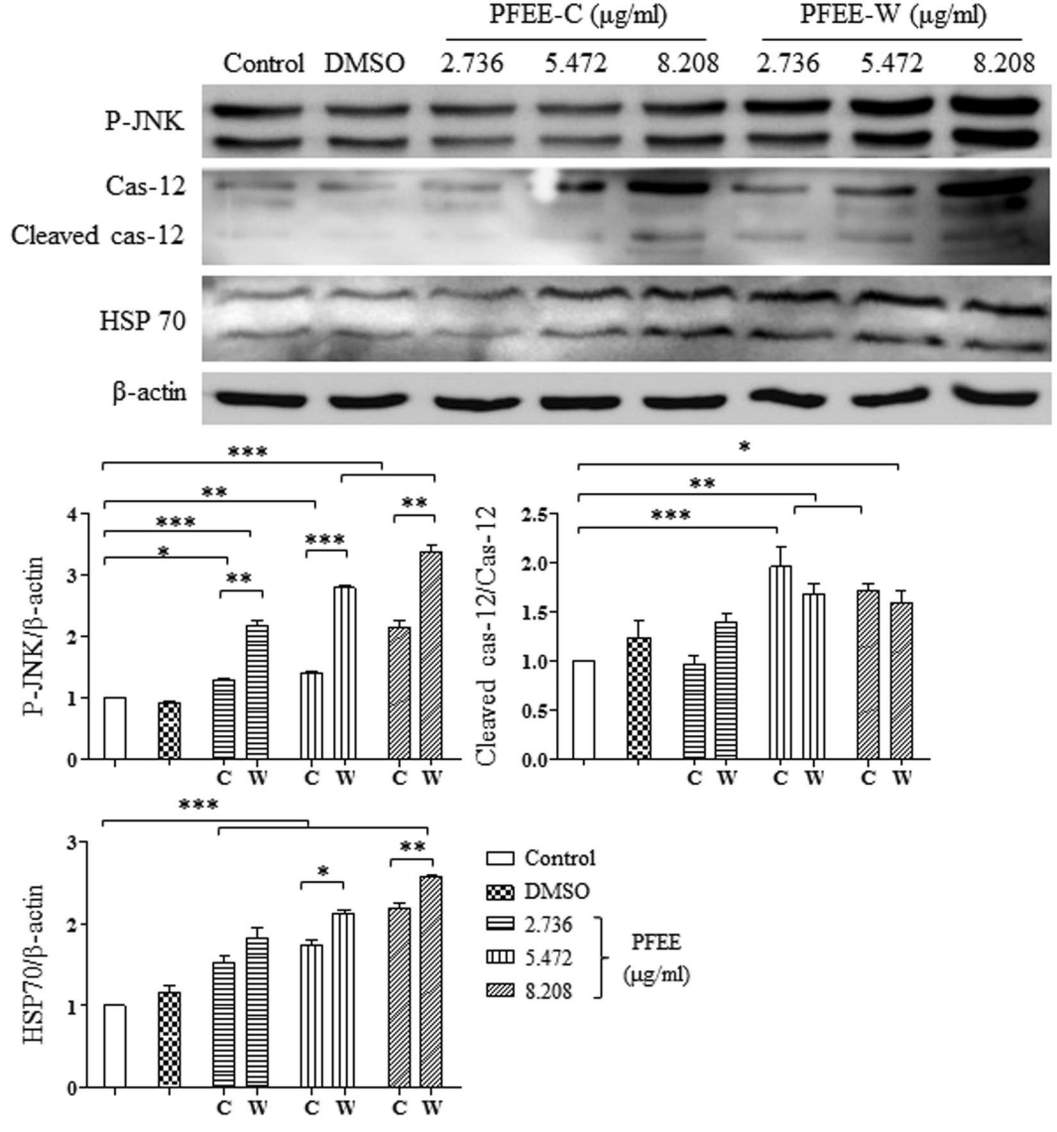
PFEE-C and PFEE-W induce ER stress in H22 cells. H22 cells were treated with PFEE-C and PFEE-W. After 24 h, proteins were isolated and the levels of ER stress-related proteins were detected by Western blot. Cropped blots are shown and full-length blots are included in the Supplementary Information. Grayscale scanning data were obtained by Image J. The ratios of HSP70/β-actin, P-JNK/β-actin and cleaved-cas-12 /cas-12 were shown in lower panels. Data are from 3 independent experiments and analyzed by ANOVA. *p < 0.05; **p < 0.01; ***p < 0.001 compared to untreated group.

It has been reported that the mitochondria-dependent apoptotic pathway can be activated by ER stress^20–22^. We explored whether the mitochondria-dependent apoptosis of H22 cells induced by PFEE-C and PFEE-W was mediated by ER stress. After PFEE-C and PFEE-W treatment for 24 h, the levels of several markers of ER stress were detected by Western blot. We found that the levels of phosphorylated c-Jun N-terminal kinase (P-JNK), cas-12, cleaved cas-12 and heat shock protein (HSP) 70 were significantly increased (p < 0.05, Fig. 6). Moreover, the level of P-JNK induced by PFEE-W was significantly higher than that of PFEE-C (p < 0.01). The result indicated that PFEE-C and PFEE-W might induce mitochondria-dependent apoptosis in H22 cells through ER stress.

### PFEE-C and PFEE-W promote reactive oxygen species (ROS) production in H22 cells

**Figure 7:**
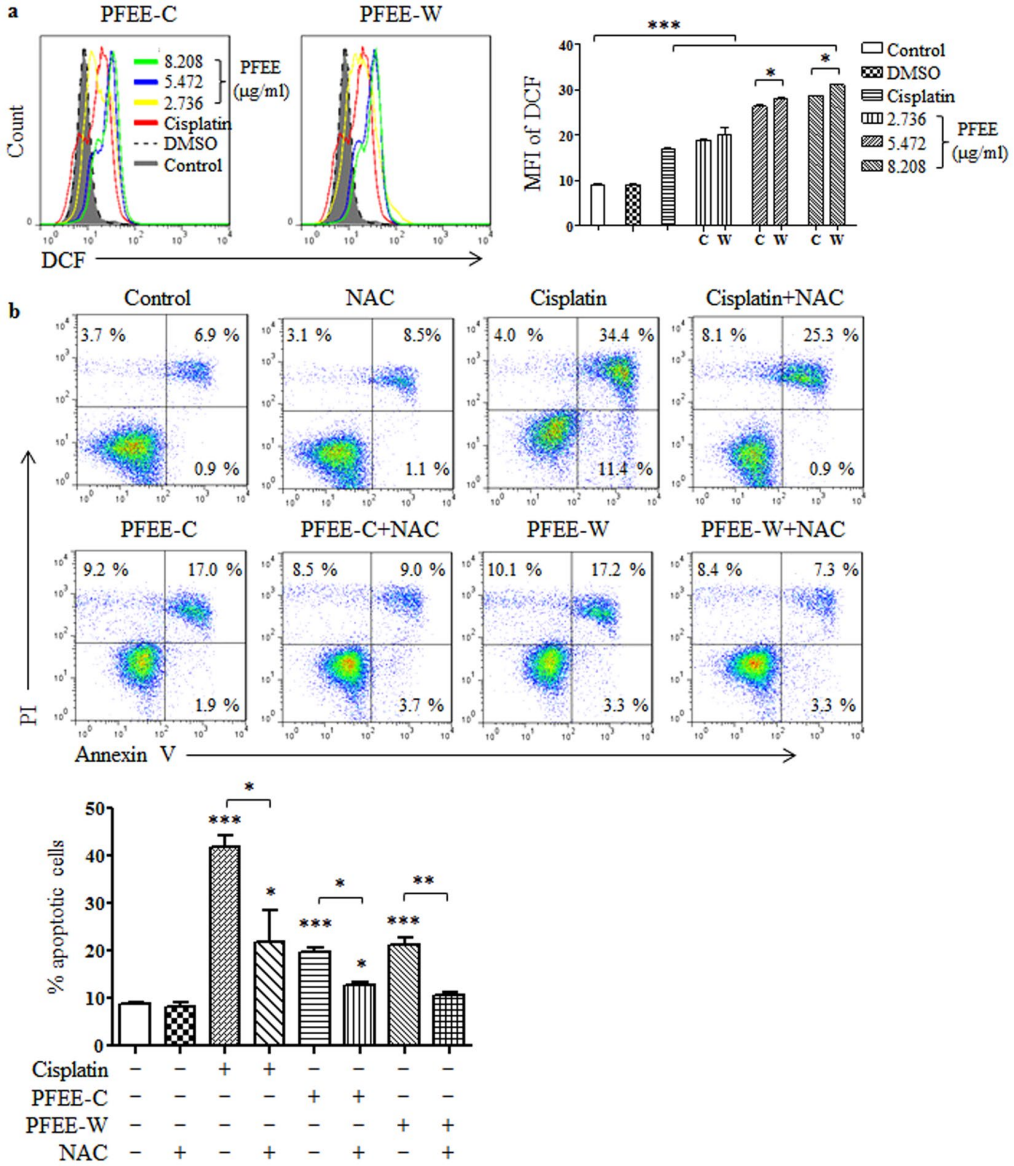
The effects of PFEE-C and PFEE-W on intracellular ROS generation in H22 cells. H22 cells were treated with different concentrations of PFEE-C and PFEE-W. (**a**) After 48 h, cells were stained with DCFH-DA and samples were analyzed by flow cytometry. (**b**) The cells were pretreated with or without 10 mM NAC for 2 h, and treated with PFEE-C and PFEE-W for 24 h, then the apoptosis of cells was analyzed by flow cytometry. Data are from 3 independent experiments and analyzed by ANOVA. *p < 0.05; **p < 0.01; ***p < 0.001 compared to untreated group.

Several studies have been shown that ROS production can induce ER stress and mitochondrial dysfunction to cause apoptosis^21,23,24^. H22 cells were treated with PFEE-C and PFEE-W for 48 h and the intracellular ROS levels were detected. As shown in Fig. 7a, the intracellular ROS levels were significantly increased by PFEE-C and PFEE-W treatment in a dose-dependent manner. We further investigated the role of ROS in the induction of apoptosis using N-acetyl-L-cysteine (NAC), a ROS scavenger. As shown in Fig. 7b, NAC pretreatment significantly inhibited the apoptosis of H22 cells induced by PFEE-C and PFEE-W, suggesting that PFEE-induced apoptosis in H22 cells was partially depend on the ROS production.

### PFEE-C and PFEE-W inhibit H22 cell migration *in vitro*

**Figure 8:**
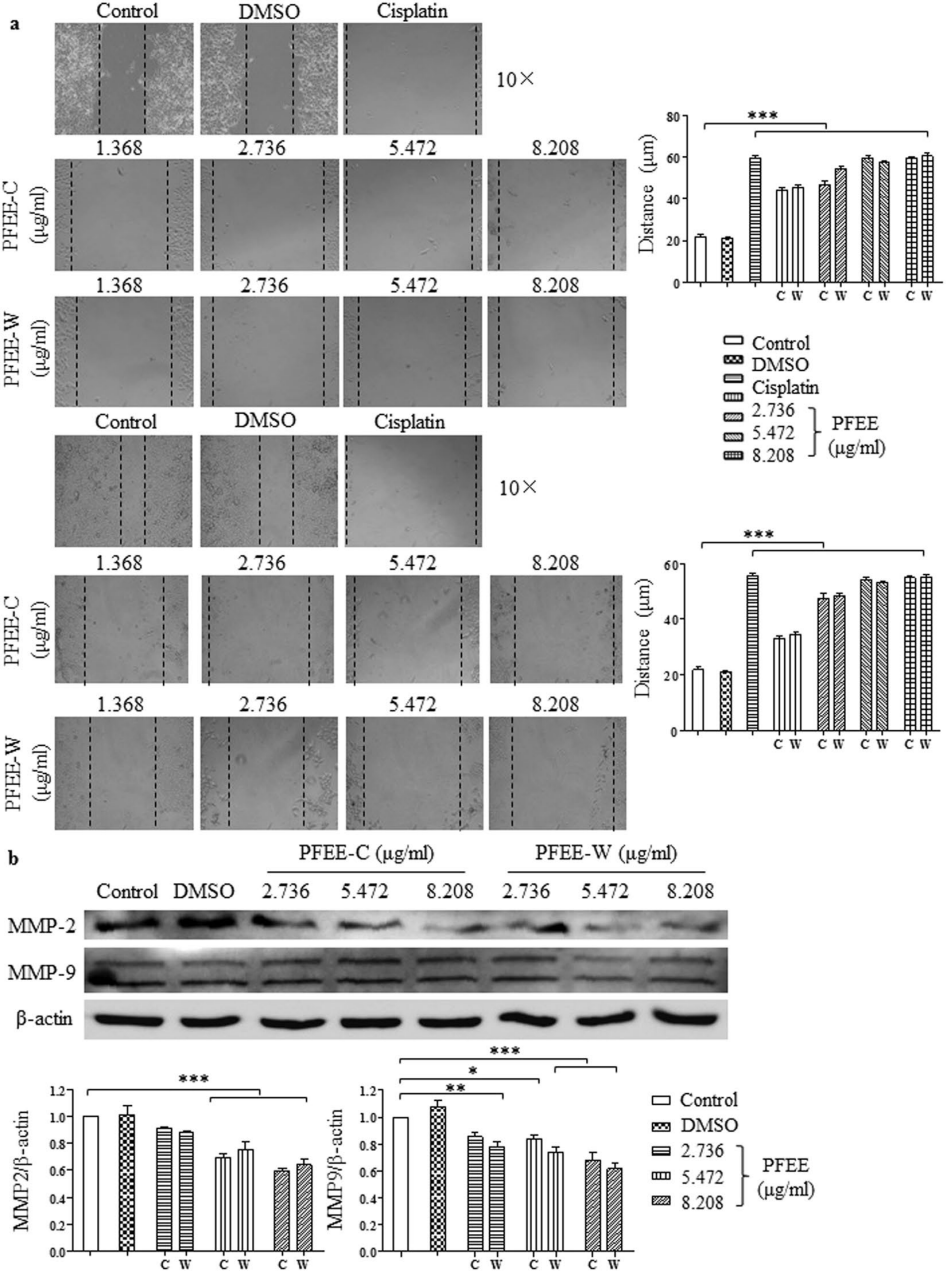
PFEE-C and PFEE-W inhibited H22 cell migration *in vitro*. (**a**) After PFEE-C and PFEE-W treatment for 24 h and 48 h, H22 cell migration was observed by inverted microscope and analyzed by Image J. The width of scratches was shown in lower panels. (**b**) After PFEE-C and PFEE-W treatment for 24 h, proteins were isolated from H22 cells to detect the levels of MMP-2 and MMP-9 by Western blot. Cropped blots are shown and full-length blots are included in the Supplementary Information. The ratios of MMP-2/β-actin and MMP-9/β-actin were shown in lower panels. Data were analyzed by ANOVA. *p < 0.05; **p < 0.01; ***p < 0.001 compared to untreated group.

To determine whether PFEE-C and PFEE-W affect H22 cell migration, the center of the culture dishes was scratched using 200 μl pipette when H22 cells grew to 80% confluency. Then, these samples were treated with different concentrations of PFEE-C and PFEE-W for 48 h. The pictures were taken by inverted microscope at 24 and 48 h, respectively, and the width of scratches was analyzed by Image J. As shown in Fig. 8a, H22 cell migration was significantly inhibited by PFEE-C and PFEE-W treatment in dose-dependent manner (p < 0.001). Matrix metalloproteinase (MMP) family plays a critical role in the migration of tumor cells^25^. After PFEE-C and PFEE-W treatment for 24 h, the levels of MMP-2 and MMP-9 were significantly decreased (p < 0.05, Fig. 8b), suggesting that PFEE-C and PFEE-W might suppress the invasion and metastasis of HCC.

### PFEE-C suppresses H22 cell growth *in vivo*

**Figure 9:**
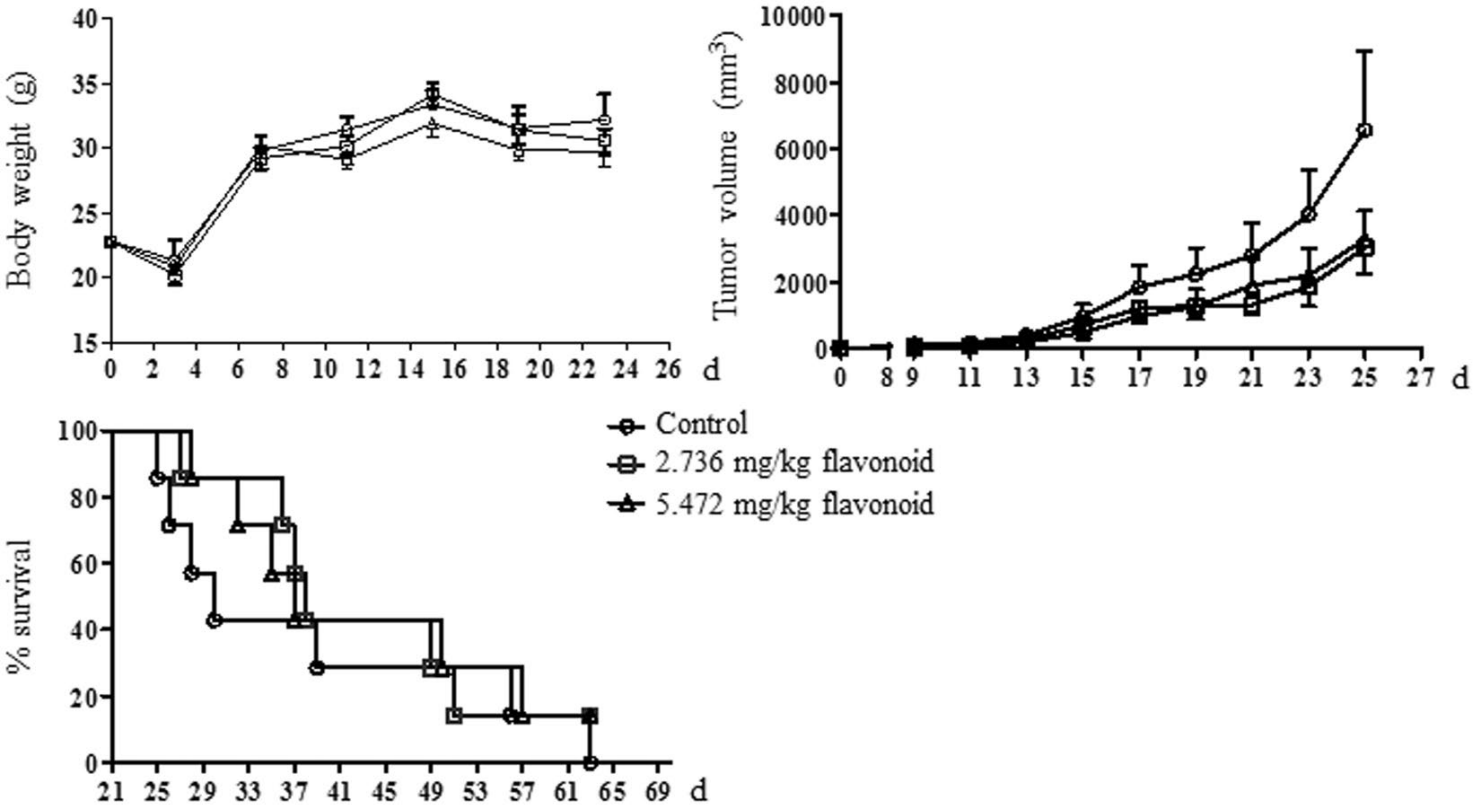
The inhibition of tumor growth *in vivo*. Tumor mouse model was induced by injection of H22 cells. After 3 days, tumor mice (7 mice per group) were treated with or without PFEE-C. Body weight, tumor volume and survival rate were monitored at the indicated time points.

We further examined the effect of PFEE-C on H22 cell growth *in vivo*. 1 × 10^6^ H22 cells were injected into the right flank of Kunming male mice. After 3 days, tumor mice were treated with DMSO (control), 2.74 or 5.48 mg/kg flavonoids of PFEE-C around tumor every other day for 7 times. As shown in Fig. 9, body weight of tumor mice had no significant difference. However, the tumor volumes (2782 mm^3^ and 3014 mm^3^) of groups treated with 2.74 or 5.48 mg/kg flavonoids were greatly inhibited compared with DMSO group (6594 mm^3^). At the end of this tumor study, the survival rate was calculated. On day 30, the survival rates of groups treated with DMSO, 2.74 or 5.48 mg/kg flavonoids were 42.9%, 85.7% and 85.7%, respectively. On day 62, all mice were dead in DMSO group and 1 tumor mouse in each PFEE-C group survived. The results suggested that PFEE-C suppressed the tumor growth and increased the survival rate.

## Discussion

In this study, we found that both PFEE-C and PFEE-W inhibited the growth of H22 cells through induction of apoptosis via mitochondria-dependent pathway, suppressed the migration of H22 cells by the down-regulation of MMP-2 and MMP-9, and induced ROS production and ER stress. PFEE-W showed higher antitumor activity than PFEE-C at the same concentration of flavonoids.

It has reported that traditional Chinese medicine can induce tumor cell apoptosis through both death receptor-mediated and mitochondria-dependent pathways^26–28^. The BCL-2 protein family plays a critical role in the regulation of mitochondrial membrane integrity^18,19^. Here, we observed that PFEE-C and PFEE-W increased the ratio of Bax/Bcl-2 in H22 cells that resulted in the reduction of Δψ_m_ and the release of cytochrome c. The results indicated that PFEE-C and PFEE-W induced apoptosis of H22 cells through mitochondria-dependent pathway. Similarly, our previous study reported that PFEE-C induced apoptosis of B16F10 cells via mitochondria-dependent pathway^11^.

Various factors including oxidative stress and Ca^2+^ depletion can cause ER stress that activates unfolded protein response (UPR) to restore homeostasis^29^. However, cells will activate death programs when UPR fails. Recently, a large body of evidence has been shown that ER stress plays important roles in the induction of apoptosis^20,22,30,31^, which can activate JNK, promote caspase-12 cleavage and increase HSP70 level^32–34^. JNK can regulate some BCL-2 family proteins such as phosphorylation of Bcl-2 and Bim to cause Δψ_m_ reduction, promote cytochrome c release and induce apoptosis^35,36^. The processing of caspase-12 can promote the cleavage of caspase-9 and caspase-3^37,38^. We found that PFEE-C and PFEE-W significantly up-regulated the levels of P-JNK, cleaved caspase-12 and HSP70 that might result in increasing the ratio of Bax/Bcl-2, reducing Δψ_m_, enhancing cytochrome c release and the cleavage of caspase-9 and caspase-3. These results indicated that PFEE-C and PFEE-W might lead to ER stress and mitochondrial dysfunction to induce H22 cell apoptosis. ER stress and mitochondrial dysfunction can be induced by ROS generation^21,23,24^. Our data showed that PFEE-C and PFEE-W dramatically increased the level of ROS in H22 cells, which might cause ER stress and mitochondria dysfunction.

PFEE-C was chosen to detect the antitumor effect in tumor mouse model and greatly inhibited tumor growth, but the survival rate was improved only in a narrow window period, which might be due to the early stop of drug administration. In the future study, we will prolong the drug administration to detect the effect of PFEE-C and PFEE-W on the survival of tumor mice.

In summary, PFEE-C and PFEE-W inhibited H22 cell growth through induction of ER stress- and mitochondria-mediated apoptosis that might be associated with ROS generation. However, the components of PFEE-C and PFEE-W and its antitumor mechanism need to be further investigated.

## Material and Methods

### Preparation of ethanol Extractions of wild and cultivated *P. ferulae*

Cultivated and wild *P. ferulae* were collected from Jinghe in Xinjiang Uygur Autonomous Region, China. The ethanol extractions of cultivated and wild *P. ferulae* (PFEE-C and PFEE-W) were prepared according to our previous description with some modifications^11^. Briefly, 100 g powders of wild and cultivated *P. ferulae* were extracted three times using 1 L of 95% (v/v) ethanol with stirring at 60 °C for 2 h, followed by sonication for 30 min under 300 W at 25 °C. The extracts were collected together and filtered through Whatman No. 4 filter paper after centrifugation at 5000 rpm for 10 min. Ethanol was subsequently removed from the extracts using a rotary vacuum evaporator at 45 °C, followed by a freeze-drier. Extracts were dissolved in dimethyl sulfoxide (DMSO) (Sigma, St. Louis, MO, USA) at the concentration of 100 mg/ml and filtered with a 0.22 μm filter.

### Determination of total flavonoid content

The content of flavonoids was detected according to previous description with minor modifications^39^. Briefly, 0.5 ml PFEE-C and PFEE-W were added with 30 μl of 5% NaNO_2_ for 6 min at room temperature (RT), followed by adding 30 μl of 10% Al(NO_3_)_3_ for another 6 min at RT. Then, the solution was thoroughly mixed with 0.4 mL of 1 M NaOH and 40 μl 30% ethanol and incubated for 15 min at RT. Optical densities (OD) of the mixture was detected at a wavelength of 517 nm using a 96-well microplate reader (Bio-Rad Laboratories, CA, USA). The content of flavonoids in PFEE was calculated according to a standard curve obtained by the standard of rutin.

### Cell lines and cell culture

The mouse HCC H22 cells and human HCC HepG2 cells were obtained from the Xinjiang Key Laboratory of Biological Resources and Genetic Engineering, Xinjiang University (Urumqi, Xinjiang, China) and cultured in RPMI 1640 medium (Gibco) supplemented with 10% heat-inactivated fetal bovine serum (MRC), 1% L-glutamine (100 mM), 100 U/ml penicillin and 100 μg/ml streptomycin at 37 °C in a humidified atmosphere of 5% CO_2_.

### MTT assay

The antitumor effects of PFEE-C and PFEE-W on H22 and HepG2 cells were assessed by MTT [3-(4,5-dimethyl-2- thiazolyl)-2,5- diphenyl-2-H-tetrazolium bromide] (Sigma, MO, USA) assay. H22 and HepG2 cells at the logarithmic growth phase were seeded in 96-well plates at a density of 5 × 10^3^ cells/well and cultured overnight. Cells were treated with PFEE-C and PFEE-W at various concentrations (0, 1.368, 2.736, 5.472, 8.208 μg/ml) of flavonoids or 0.6% DMSO (equal to that in the highest dose of PFEE) for 24, 48 and 72 h, respectively. Cisplatin was used as a positive control. After centrifugation at 1200 rpm for 7 min, supernatant was discarded and 100 μl of MTT solution (0.5 mg/ml in PBS) was added to each well. After incubation for 4 h at 37 °C, the formed formazan crystals were dissolved in 150 μl DMSO. The OD_490_ values were measured by a 96-well microplate reader (Bio-Rad Laboratories, CA, USA).

To evaluate the effects of PFEE-C and PFEE-W on splenocytes, cells were isolated from C57BL/6 mice and plated into 96-well plates at a density of 1 × 10^5^ cells/well. Splenocytes were treated with PFEE-C and PFEE-W according to the above concentrations for 24 h and 48 h. The relative cell viability was calculated as the followed formula: Cell viability (%) = (OD_treated_/OD_untreated_) × 100%.

### Apoptosis analysis

H22 and HepG2 cells were treated with PFEE-C and PFEE-W at various concentrations (0, 2.736, 5.472, 8.208 μg/ml) of flavonoids or 0.6% DMSO for 24 h. All cells were harvested and stained with Annexin V-FITC/propidiumidide (PI) Apoptosis Detection Kit (YEASEN, China) according to the manufacturer’s instructions. In some experiments, cells were pretreated with 10 mM N-acetyl-L-cysteine (NAC, Sigma) for 2 h, and then treated with PFEE for 24 h to detect the apoptosis. Samples were analyzed by flow cytometry (BD FACSCalibur, CA, USA).

### Hoechst 33258 staining

H22 and HepG2 cells were treated with PFEE-C and PFEE-W according to apoptosis analysis for 24 h. After washing with PBS, cells were fixed with 4% ice-cold paraformaldehyde at 4 °C for 10 min, and stained with Hoechst 33258 (Beyotime, China) at 4 °C for 10 min. Samples were observed by inverted fluorescence microscope (Nikon Eclipse Ti-E, Japan).

### Cell cycle analysis

H22 cells were treated with PFEE-C and PFEE-W according to MTT assay for 24 h. Cells were collected and washed with PBS, then fixed by 70% (v/v) ice-cold ethanol for 30 min at 4 °C. After washing twice with 5 ml PBS, cells were stained with 0.3 ml PI for 30 min at 37 °C. Samples were analyzed by flow cytometry (BD FACSCalibur, CA, USA).

### Measurement of intracellular reactive oxygen species (ROS)

Intracellular production of ROS was measured using DCFH-DA probes. H22 cells were treated with PFEE-C and PFEE-W according to apoptosis analysis for 48 h. Cells were washed with PBS and stained by 10 mM of fluorescent probe DCFH-DA (Beyotime, China) for 20 min at 37 °C. After washing three times with PBS, the fluorescence intensity in cells was determined using flow cytometry (BD FACSCalibur, CA, USA).

### Detection of mitochondrial membrane potential (Δ*ψ*_*m*_)

H22 cells were treated with PFEE-C and PFEE-W according to apoptosis analysis for 48 h. After washing twice with PBS, cells were re-suspended with 300 μl of JC-1 staining solution and incubated at 37 °C for 30 min, then observed by inverted fluorescence microscopy and analyzed by flow cytometry (BD FACSCalibur, CA, USA).

### Migration assay

H22 cells (2.5 × 10^4^/well) were seeded in a 24-well plate. After reaching 80% confluency, the center of each well was scratched once with a 200 μl pipette tip. After washing with PBS, fresh medium contained PFEE-C and PFEE-W at various concentrations (0, 1.368, 2.736, 5.472, 8.208 μg/ml) of flavonoids or 0.6% DMSO was added and incubated at 37 °C. After 48 h, images of each sample were taken under a microscope (Nikon Eclipse Ti-E, Japan). The average distances of cell migration were analyzed by Image J.

### Western blot

H22 cells were treated with PFEE-C and PFEE-W according to apoptosis analysis for 24 h. After washing with ice-cold PBS twice, cells were collected and lysed in RIPA Lysis Buffer (Beijing ComWin Biotech Co., Ltd) for 20 min on ice. After centrifugation at 12,000 rpm 4 °C for 10 min, the protein concentration in supernatant was determined by the bicinchoninic acid assay kit (Thermo Fisher Scientific, USA). Proteins at same concentration were separated by 12% SDS-PAGE and transferred to PVDF membranes. After washing with PBST buffer (PBS with 0.05% Tween-20), membrane was blocked with 5% skim milk at 37 °C for 1 h, and then incubated with the primary antibodies (Cell Signaling Technology, MA, USA) at proper dilutions overnight at 4 °C. After washing three times with PBST, membrane was incubated with the corresponding HRP-conjugated secondary antibodies (Cell Signaling Technology) for 2 h at 37 °C. The target proteins were detected using ECL assay kit (Beyotime, China).

### Animals and ethics statement

Kunming male mice (6–8 weeks age) were purchased from Animal Laboratory Center, Xinjiang Medical University (Urumqi, Xinjiang, China) and housed in a temperature-controlled, light-cycled animal facility of Xinjiang University. All animal experiments were approved by the Committee on the Ethics of Animal Experiments of Xinjiang Key Laboratory of Biological Resources and Genetic Engineering (BRGE-AE001) and performed under the guidelines of the Animal Care and Use Committee of College of Life Science and Technology, Xinjiang University.

### *In vivo* tumor study

H22 cells (1 × 10^6^ cells/mice) were subcutaneously injected into the flanks of Kunming mice. Tumor mice were randomly divided into 3 groups (7 mice/group). After 3 days, tumor mice were treated with 0.1 ml DMSO, PFEE-C with 2.736 mg/kg flavonoids in 0.1 ml DMSO or 5.472 mg/kg flavonoids in 0.1 ml DMSO around tumor. Mice were treated every 2 days for up to 15 days. Tumor sizes were measured using calipers and tumor volumes were calculated according to the following formula: tumor volume (mm^3^) = (length × width^2^)/2. At the end of this study (on day 62), the survival rates of tumor mice in each group were calculated with Prism 5.

### Statistical Analysis

All data were expressed as mean ± standard error of the mean (SEM). Statistical analysis was conducted using one-way analysis of variance (ANOVA). The paired two-tailed t test was used for comparing PFEE-C and PFEE-W. *p* < 0.05 was considered statistically significant.

## Electronic supplementary material

Supplemental figures
