# Supplementary material for: Cultivated and wild Pleurotus ferulae ethanol extracts inhibit hepatocellular carcinoma cell growth via inducing endoplasmic reticulum stress- and mitochondria-dependent apoptosis
Source: Sci Rep. 2018 Sep 18;8:13984. doi: 10.1038/s41598-018-32225-4 (PMC6143524; doi:10.1038/s41598-018-32225-4)
Supplement: Supplementary file 1 — Supplemental figures [file 41598_2018_32225_MOESM1_ESM.docx]

**Cultivated and wild *Pleurotus ferulae* ethanol extracts inhibit hepatocellular carcinoma cell growth via inducing** **endoplasmic reticulum stress- and mitochondria-dependent apoptosis**

Yi Yang^a^, Pengfei Yuan^a^, Xianxian Wei^a^, Changshuang Fu^a^, Jinyu Li^b*^, Weilan Wang^a^, Xinhui Wang^a^, Yijie Li^a^, Jinyao Li^a,c,^*

^a^ Xinjiang Key Laboratory of Biological Resources and Genetic Engineering, College of Life Science and Technology, Xinjiang University, Urumqi 830046, China;

^b^ College of Life Science, Xinjiang Normal University, Urumqi 830054, China

^c^ Affliated Tumor Hospital of Xinjiang Medical University, Urumqi 830011, China

^*^ Corresponding Authors: Jinyao Li, College of Life Science and Technology, Xinjiang University, 666 Shengli Road, Urumqi 830046, China. Tel.: +86-991-8583259; Fax: +86-991-8583517, e-mail: ljyxju@xju.edu.cn and Jinyu Li, College of Life Science, Xinjiang Normal University, 102 Xinyi Road, Urumqi 830054, China. Tel.: +86-991-4332474; e-mail: lijinyu234@163.com

**
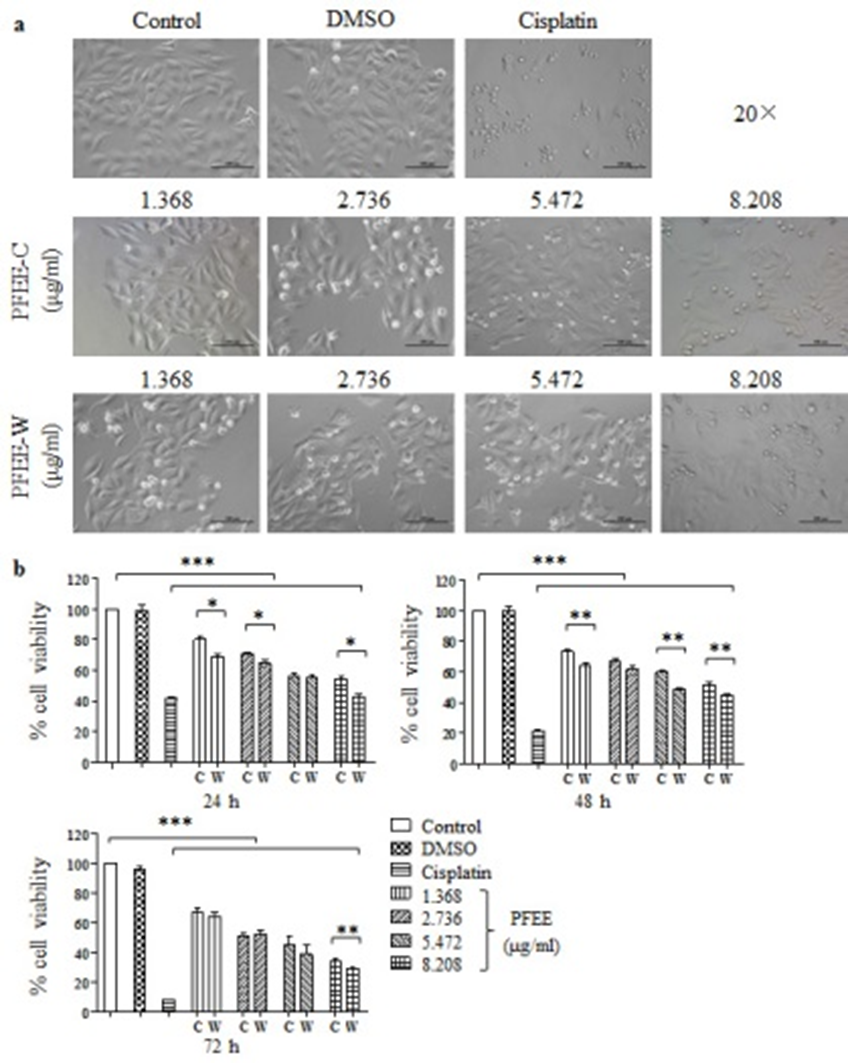
**

**Supplemental Figure 1. Effects of PFEE-C and PFEE-W on the proliferation of HepG2 cells.** (a) The morphological changes of HepG2 cells after PFEE-C and PFEE-W treatment for 24 h. (b) The viability of HepG2 cells after PFEE-C and PFEE-W treatment for 24, 48 and 72 h, respectively. Data are from 3 independent experiments and analyzed by ANOVA. * *p* < 0.05; ** *p* < 0.01; *** *p* < 0.001 compared to untreated group.

**
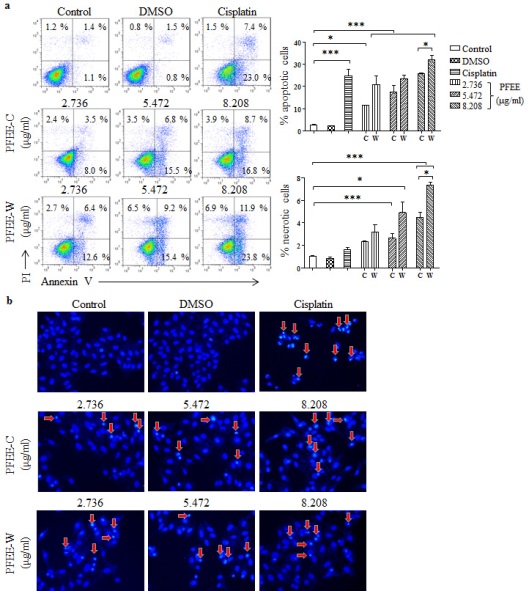
**

**Supplemental Figure 2. The apoptosis of HepG2 cells induced by PFEE-C and PFEE-W treatment.** Different concentrations of PFEE-C and PFEE-W were used to treat HepG2 cells for 24 h. (a) The apoptosis and necrosis of HepG2 cells were analyzed by flow cytometry. The individual dot plots were shown in left panels and the summary data were shown in right panels. (b) The nuclear morphology of HepG2 cells. The above HepG2 cells were stained with Hoechst 33258 and observed by inverted fluorescence microscopy. The arrows indicated the chromosomal condensation. Data are from 3 independent experiments and analyzed by ANOVA. * *p* < 0.05; *** *p* < 0.001 compared to untreated group.
